# Evidence of bendamustine plus rituximab for old and frail patients with aggressive B-cell lymphoma

**DOI:** 10.1007/s00277-023-05166-w

**Published:** 2023-03-16

**Authors:** Enrico Schalk, Kathleen Jentsch-Ullrich

**Affiliations:** 1 Department of Hematology and Oncology, Medical Center, Otto-von-Guericke University Magdeburg, Leipziger Str. 44, 39120 Magdeburg, Germany; 2 Practice for Hematology and Oncology, Magdeburg, Germany

Dear Editor,

Recently, the largest prospective cohort of old and frail patients with aggressive B-cell lymphoma and bendamustine/rituximab (BR) as first-line treatment was reported (B-R-ENDA trial) [1]. B-R-ENDA was a multi-center, prospective, non-randomized trial on patients > 80 or 61–80 years not qualifying for CHOP (cyclophosphamide, doxorubicin, vincristine, prednisolone)-like therapy. Sixty-eight patients were included, with 39 patients (57%) > 80 years (target cohort; Table 1). However, due to slow recruitment, the trial was terminated before the planned target (50 patients > 80 years) was reached [1]. In line with four other bendamustine-based prospective trials (with only 14–49 patients) [1], it shows the difficulty of performing clinical trials in this patients’ population. These difficulties and the fact that standard treatment for old or frail patients not eligible for CHOP(-like) therapy has not been defined [1], retrospective studies are useful to answer important clinical questions [2].

**Table 1:** Comparison of R-Benda study and B-R-ENDA trial

	R-Benda study *n* = 68			B-R-ENDA trial *n* = 68			
**General study overview**							
Publication year	2019			2022			
Study period	February 2008–September 2017			July 2012–February 2016			
Study sites, n, country	4, Germany			24, Germany			
Study design	Retrospective			Prospective, phase 2			
Included pathological diagnosis	Diffuse large B-cell lymphoma			CD20 + aggressive B-cell lymphoma			
Population/Subgroups	> 80 years *n* = 28	61–80 years *n* = 40	Total *n* = 68	> 80 years *n* = 39	61–80 years *n* = 29	Total *n* = 68	*P* value
Patients’ characteristics							
Females, n/N (%)	10/28 (36)	23/40 (58)	33/68 (49)	28/39 (72)	18/29 (62)	46/68 (68)	0.007* 0.90** 0.04***
Age, median, years (range)	83.5 (81–91)	77.5 (68–80)	80 (68–91)	84 (81–95)	77 (64–80)	81 (64–95)	n/a
Age groups, n/N (%)							
61–75 years	-	7/40 (18)	7/68 (10)	-	8/29 (28)	8/68 (12)	0.48** 1.00***
76–80 years	-	33/40 (83)	33/68 (49)	-	21/29 (72)	21/68 (31)	0.48** 0.05***
81–85 years	22/28 (79)	-	22/68 (32)	25/39 (64)	-	25/68 (37)	0.31* 0.72***
> 85 years	6/28 (21)	-	6/68 (9)	14/39 (36)	-	14/68 (21)	0.31* 0.09***
ECOG PS > 1, n/N (%)	8/26 (31)	16/39^a^ (41)	24/65^a^ (37)	9/39 (23)	15/29 (52)	24/68 (35)	0.68* 0.53** 0.99***
Stage III/IV, n/N (%)	20/28 (71)	28/40 (70)	48/68 (71)	20/39 (51)	19/29 (66)	39/68 (57)	0.16* 0.89** 0.15***
Extranodal involvement, n/N (%)	24/28 (86)	31/40 (78)	55/68 (81)	21/39 (54)	19/29 (66)	40/68 (59)	0.01* 0.41** 0.008***
IPI score (risk), n/N (%)							
1 (low)	2/25^a^ (8)	1/37^a^ (3)	3/62^a^ (5)	6/39 (15)	2/29 (7)	8/68 (12)	0.64* 0.82** 0.27***
2 (low-intermediate)	5/25^a^ (20)	9/37^a^ (24)	14/62^a^ (23)	15/39 (38)	5/29 (17)	20/68 (29)	0.20* 0.70** 0.49***
3 (high-intermediate)	7/25^a^ (28)	11/37^a^ (30)	18/62^a^ (29)	11/39 (28)	12/29 (41)	23/68 (34)	1.00* 0.47** 0.69***
4–5 (high)	11/25^a^ (44)	16/37^a^ (43)	27/62^a^ (44)	7/39 (18)	10/29 (34)	17/68 (25)	0.05* 0.64** 0.04***
Bulky disease, n/N (%)	11/28 (39)	10/39^a^ (26)	21/67^a^ (31)	8/39 (21)	11/29 (38)	19/68 (28)	0.16* 0.41** 0.81***
Bone marrow involvement, n/N (%)	1/22^a^ (5)	7/38^a^ (18)	8/60^a^ (13)	0	2/29 (7)	2/68 (3)	0.72* 0.31** 0.06***
Diffuse large B-cell lymphoma, n/N (%)	28/28 (100)	40/40 (100)	68/68 (100)	31/39 (79)	26/29 (90)	57/68 (84)	-
Treatment response, n/N (%)							
Complete remission	11/28 (39)	17/39^a^ (44)	28/68 (38)	18/39 (46)	3/29 (10)	21/68 (31)	0.76* 0.005** 0.28***
Partial remission	5/28 (18)	9/39^a^ (23)	14/68 (21)	2/39 (5)	5/29 (17)	7/68 (10)	0.20* 0.78** 0.15***
Stable disease	0	1/39^a^ (3)	1/68 (1)	2/39 (5)	1/29 (3)	3/68 (4)	-
Progressive disease	2/28 (7)	5/39^a^ (13)	7/68 (10)	5/39 (13)	9/39 (31)	14/68 (21)	-
Outcome							
Follow-up, median, months (95% CI)	48 (17–78)	44 (18–70)	48 (28–67)	29 (n/a)	27 (n/a)	n/a	-
PFS, median, months (95% CI)	8 (2–14)	12 (0–29)	11 (5–17)	13 (n/a)	5 (n/a)	n/a	-
OS, median, months (95% CI)	13 (6–20)	30 (19–42)	16 (11–22)	16 (n/a)	14 (n/a)	n/a	-
2-year PFS, % (95% CI)	21 (5–38)	46 (30–62)	36 (24–48)	45 (28–61)	32 (13–51)	40 (27–52)	-
2-year OS, % (95% CI)	21 (5–37)	55 (39–72)	41 (28–53)	46 (28–63)	37 (17–57)	42 (29–55)	-

*ECOG PS*, Eastern Cooperative Oncology Group performance score; *IPI*, International Prognostic Index; *n/a*, not available; *OS*, overall survival; *PFS*, progression-free survival; *95%CI*, 95% confidence interval

^a^Partially missing data

*Comparison of cohort > 80 years in R-Benda study *vs.* B-R-ENDA trial

**Comparison of cohort 61–80 years in R-Benda study *vs.* B-R-ENDA trial

***Comparison of all patients in the R-Benda study *vs.* B-R-ENDA trial

If applicable, Fisher’s exact test was used to compare patients’ characteristics and treatment response between R-Benda and B-R-ENDA. The Kaplan–Meier method was used for survival analyses. Statistical analysis was performed using OpenEpi, version 3.01 (Atlanta, GA, USA, https://www.openepi.com), and IBM® SPSS® Statistics, version 28 (Armonk, NY, USA). Two-sided *P* values < 0.05 were considered statistically significant

To build more evidence on BR for older patients with aggressive B-cell lymphoma and to compare the results of a prospective clinical trial with real-world data, the aim was to compare the B-R-ENDA data with another study. However, it is difficult to compare different studies, e.g., multi-center *vs.* single-center analysis, prospective *vs.* retrospective design, differences in inclusion/exclusion criteria, study endpoints, or sample sizes. A retrospective study on 68 patients treated with BR for aggressive B-cell lymphoma (R-Benda study) [3] is useful for comparison with B-R-ENDA because that was also a multi-center study with the same sample size.

R-Benda was conducted on older or frail patients ≥ 65 years with ECOG performance score (PS) ≥ 2 or ≥ 75 years regardless of ECOG PS with de novo diffuse large B-cell lymphoma (DLBCL). The aim of this study was to compare the outcome of patients treated with BR *vs.* rituximab/CHOP. Sixty-eight patients treated with BR were analyzed within this study [3].

For direct comparison of both R-Benda and B-R-ENDA, the same parameters and endpoints, respectively, need to be analyzed. Therefore, the original R-Benda data set had to be re-evaluated according to the B-R-ENDA analyses (Table 1).

Regarding age, the whole R-Benda cohort was comparable with the whole B-R-ENDA cohort (median age 80 *vs.* 81 years). There were also no statistical differences in the portion of patients > 80 years (41% *vs.* 57%; *P* = 0.09). However, in R-Benda, in the subgroup of > 80 years (and the whole R-Benda cohort), there were fewer females than in B-R-ENDA. Regarding lymphoma specific parameters, in R-Benda, more patients > 80 years (and in the whole R-Benda cohort) had extranodal involvement compared to B-R-ENDA. According to the International Prognostic Index (IPI) score, in R-Benda, at least numeric more patients > 80 years (and in the whole cohort) had high-risk disease than in B-R-ENDA. The remission rates in the subgroup > 80 years (and in the whole cohort) were comparable between R-Benda and B-R-ENDA (Table 1). However, the overall response rate (sum of complete and partial remission) in the whole R-Benda cohort was higher compared to the whole B-R-ENDA cohort (62% *vs.* 41%; *P* = 0.03), caused by the higher complete remission (CR) rate in the cohort 61–80 years in R-Benda (44% *vs.* 10%; *P* = 0.005). The lower CR rate in B-R-ENDA, however, was expected based on the inclusion criteria for this age group [1].

The primary endpoint in B-R-ENDA was progression-free survival at 2 years (2y-PFS) [1]. After re-assessment of the R-Benda data set, it was possible to provide also the 2y-PFS rate. In the subgroup > 80 years, the 2y-PFS in R-Benda was markedly inferior compared to B-R-ENDA (21% *vs.* 45%), but less pronounced for the whole cohorts (36% *vs.* 40%). The same was true for 2-year overall survival rates, even for the whole cohorts the rates were equal (41% *vs.* 42%).

R-Benda and B-R-ENDA included older (≥ 75 years or > 80 years, respectively) or frail patients (≥ 65 years plus ECOG PS ≥ 2 or 61–80 years plus Cumulative Illness Rating Scale [CIRS] score > 6, respectively). Regarding ECOG PS, there were no differences between both studies. In R-Benda, the CIRS score was not considered, but the Charlson Comorbidity Index (CCI). CCI and CIRS score correlate in cancer patients [4, 5]. In B-R-ENDA, 72% of the whole cohort had a CIRS score > 6 indicating medical non-fit patients [1]. In the whole R-Benda cohort, 82% of the patients had an age-adjusted CCI ≥ 4 indicating intermediate-high risk patients [3], corresponding to medical non-fit patients. Therefore, taking age, CCI/CIRS score, and ECOG PS into account, the patients’ characteristics of the both cohorts were comparable. The survival differences between both studies may be attributed to sex differences, because the prognosis in patients with rituximab-based regimens for DLBCL is inferior in men [6]. High-risk disease according to IPI score as well as extranodal site involvement are also independent risk factors for survival [1, 7]. Because in R-Benda, at least for patients > 80 years, more men, more patients with high IPI score, and more extranodal sites involvement were included, the inferior survival can be explained.

Although patients in clinical trials often differ substantially from those in the general population, that was not true for R-Benda. Hence, daily clinical practice matches clinical trial data (Supplementary Table 1) [1, 8]—or vice versa—in this high-risk population. The results of the re-analysis of R-Benda confirm the 2y-PFS as a surrogate endpoint [1, 8, 9].

## Supplementary Information

Below is the link to the electronic supplementary material.

Supplementary file1 (DOCX 46 KB)

## Data Availability

The data that support the findings of this study are available from the corresponding author upon reasonable request.
