# Supplementary material for: Evidence of bendamustine plus rituximab for old and frail patients with aggressive B-cell lymphoma
Source: Ann Hematol. 2023 Mar 16;102(6):1617–20. doi: 10.1007/s00277-023-05166-w (PMC10182158; doi:10.1007/s00277-023-05166-w)
Supplement: Supplementary file 1 — Supplementary file1 (DOCX 46 KB) [file 277_2023_5166_MOESM1_ESM.docx]

**Supplementary Table 1.** Descriptive comparison of R-Benda study, B-R-ENDA trial and FIL trial

|  | **R-Benda study^1^**  **n=68, in total** | **B-R-ENDA trial^2^**  **n=68, in total** | **FIL trial^3^**  **n=45, in total** |
| --- | --- | --- | --- |
| **General study overview** |  |  |  |
| Publication year | 2019 | 2022 | 2018 |
| Study period | February 2008 – September 2017 | July 2012 – February 2016 | February 2012 – February 2014 |
| Study sites, n, country | 4, Germany | 24, Germany | 24, Italy |
| Study design | Retrospective | Prospective, phase 2 | Prospective, phase 2 |
| Included pathological diagnosis | Diffuse large B-cell lymphoma | CD20+ aggressive B-cell lymphoma | Diffuse large B-cell lymphoma |
|  |  |  |  |
| **Patients’ characteristics** |  |  |  |
| Females, n/N (%) | 33/68 (49) | 46/68 (68) | 19/45 (42) |
| Age, median, years (range) | 80 (68-91) | 81 (64-95) | 81 (71-89) |
| Age groups, n/N (%) |  |  |  |
| 61-75 years | 7/68 (10) | 8/68 (12) | n/a |
| 76-80 years | 33/68 (49) | 21/68 (31) | n/a |
| 81-85 years | 22/68 (32) | 25/68 (37) | n/a |
| >85 years | 6/68 (9) | 14/68 (21) | n/a |
| ECOG PS >1, n/N (%) | 24/65^a^ (37) | 24/68 (35) | 16/44^a^ (36) |
| Stage III/IV, n/N (%) | 48/68 (71) | 39/68 (57) | 28/45 (62) |
| Extranodal involvement, n/N (%) | 55/68 (81) | 40/68 (59) | 11/45^b^ (24) |
| IPI score (risk), n/N (%) |  |  |  |
| 1 (low) | 3/62^a^ (5) | 8/68 (12) | n/a |
| 2 (low-intermediate) | 14/62^a^ (23) | 20/68 (29) | n/a |
| 3 (high-intermediate) | 18/62^a^ (29) | 23/68 (34) | 25/44^a,c^ (57) |
| 4-5 (high) | 27/62^a^ (44) | 17/68 (25) |  |
| Bulky disease, n/N (%) | 21/67^a^ (31) | 19/68 (28) | n/a |
| Bone marrow involvement, n/N (%) | 8/60^a^ (13) | 2/68 (3) | n/a |
| Diffuse large B-cell lymphoma, n/N (%) | 68/68 (100) | 57/68 (84) | 45/45 (100) |
|  |  |  |  |
| **Treatment response, n/N (%)** |  |  |  |
| Complete remission | 28/68 (38) | 21/68 (31) | 24/45 (53) |
| Partial remission | 14/68 (21) | 7/68 (10) | 4/45 (9) |
| Stable disease | 1/68 (1) | 3/68 (4) | 1/45 (2) |
| Progressive disease | 7/68 (10) | 14/68 (21) | 13/45 (29) |
|  |  |  |  |
| **Outcome** |  |  |  |
| Follow-up, median, months (95%CI) | 48 (28-67) | n/a | 33 (n/a) |
| PFS, median, months (95%CI) | 11 (5-17) | n/a | 10 (7-25) |
| OS, median, months (95%CI) | 16 (11-22) | n/a | 30 (10-n/a) |
| 2-year PFS, % (95%CI) | 36 (24-48) | 40 (27-52) | 38 (24-51) |
| 2-year OS, % (95%CI) | 41 (28-53) | 42 (29-55) | 51 (35-65) |

ECOG PS, Eastern Cooperative Oncology Group performance score; FIL, Fondazione Italiana Linfomi; International Prognostic Index; n/a, not available; OS, overall survival; PFS, progression-free survival; 95%CI, 95% confidence interval.

^a^Partially missing data.

^b^>1 site.

^c^IPI 3-5.

**References**

1. Zeremski V, Jentsch-Ullrich K, Kahl C, Mohren M, Eberhardt J, Fischer T, Schalk E (2019) Is bendamustine-rituximab a reasonable treatment in selected older patients with diffuse large B cell lymphoma? Results from a multicentre, retrospective study. Ann Hematol 98:2729-2737. https://doi.org/10.1007/s00277-019-03819-3.

2. Braulke F, Zettl F, Ziepert M, Viardot A, Kahl C, Prange-Krex G, Korfel A, Dreyling M, Bott A, Wedding U, Reichert D, de Wit M, Hartmann F, Poeschel V, Schmitz N, Witzens-Harig M, Klapper W, Rosenwald A, Wulf G, Altmann B, Trümper L (2022) First-line treatment with bendamustine and rituximab for old and frail patients with aggressive lymphoma: results of the B-R-ENDA trial. Hemasphere 6:e808. https://doi.org/10.1097/HS9.0000000000000808.

3. Storti S, Spina M, Pesce EA, Salvi F, Merli M, Ruffini A, Cabras G, Chiappella A, Angelucci E, Fabbri A, Liberati AM, Tani M, Musuraca G, Molinari A, Petrilli MP, Palladino C, Ciancia R, Ferrario A, Gasbarrino C, Monaco F, Fraticelli V, De Vellis A, Merli F, Luminari S (2018) Rituximab plus bendamustine as front-line treatment in frail elderly (>70 years) patients with diffuse large B-cell non-Hodgkin lymphoma: a phase II multicenter study of the Fondazione Italiana Linfomi. Haematologica 103:1345-1350. https://doi.org/10.3324/haematol.2017.186569.
